# Clinical and hematological profile of patients with philadelphia-negative myeloproliferative neoplasms: First report from the Ecuadorian registry

**DOI:** 10.1016/j.htct.2026.106437

**Published:** 2026-03-13

**Authors:** C Freire, A Noboa, G Acosta, C León, H Chiang-Wong, M Buenaño, R Loachamin, P Santana, F Huamán-Garaicoa

**Affiliations:** a Centro Hemato-Oncológico Quality of Care, Guayaquil, Ecuador; b Hospital de SOLCA, Guayaquil, Ecuador; c Center for Statistical Studies and Research, Escuela Superior Politécnica del Litoral, Guayaquil, Ecuador; d Hospital del IESS Guayaquil, Ecuador; e Hospital “José Carrasco Arteaga”, Cuenca, Ecuador; f Hospital de SOLCA Quito, Ecuador; g Hospital de Especialidades “Carlos Andrade Marín” IESS Quito, Ecuador; h Faculty of Medical Sciences, Universidad Católica de Santiago de Guayaquil, Ecuador

**Keywords:** Philadelphia-negative myeloproliferative neoplasms, Polycythemia vera, Essential thrombocythemia, Primary myelofibrosis, JAK2 V617F mutation, Treatment, Survival

## Abstract

**Introduction:**

Philadelphia-negative myeloproliferative neoplasms are clonal blood disorders characterized by abnormal blood cell production. This study explores the clinical and epidemiological profiles of 111 Ecuadorian patients diagnosed with Philadelphia-negative myeloproliferative neoplasms, including polycythemia vera, essential thrombocythemia, and primary myelofibrosis, between 2014 and 2023.

**Methods:**

Patients were treated in different institutions, with clinical data collected on disease progression, complications, and survival.

**Results:**

Polycythemia vera was the most common subtype (45.9%), followed by essential thrombocythemia (42.3%) and primary myelofibrosis (9%). The *JAK2* V617F mutation was most prevalent in essential thrombocythemia (53.2%) and polycythemia vera (41.2%). Hydroxyurea, the most widely used treatment, was prescribed to 77% of the patients. Disease progression to myelofibrosis occurred in three polycythemia vera and two essential thrombocythemia cases, meanwhile One case of primary myelofibrosis and one case of myeloproliferative neoplasm, unclassified, progressed to acute myeloid leukemia. Survival rates varied across the cohort; notably, certain patients with polycythemia vera and essential thrombocythemia achieved survival durations of up to 19 years.

**Conclusion:**

These results reveal a relatively homogeneous epidemiological profile across the Latin American region and underscore the need for more multicenter studies to better characterize pH**^–^** MPNs in Ecuador and the region, to optimize diagnostic and treatment strategies.

## Introduction

Chronic myeloproliferative neoplasms (MPNs) are clonal hematopoietic disorders characterized by bone marrow proliferation of one or more myeloid lineages, resulting in an overproduction of blood cells in the peripheral blood [1]. The most clinically relevant MPNs include chronic myeloid leukemia (Philadelphia chromosome-positive) and Philadelphia-negative chronic myeloproliferative neoplasms (pH**^–^** MPNs). Three classical entities are recognized within the pH**^–^** MPN category: Polycythemia vera (PV), Essential thrombocythemia (ET), and Primary myelofibrosis (PMF).

pH**^–^** MPNs are defined by the presence of driver molecular alterations that affect the JAK-STAT signaling pathway and hematopoietic growth factor receptors. These mutations, which involve the *JAK2, CALR*, and *MPL* genes, are part of the current diagnostic criteria for these disorders and are typically assessed in peripheral blood and bone marrow samples [1,2].

In 2005, the somatic p.V617F mutation in the *JAK2* gene was first identified in patients with MPNs. The JAK2V617F mutation is detected in 90–95 % of patients with PV, 60 % of patients with ET and 60 % of patients with PMF. The presence of the JAK2V617F mutation was incorporated by the World Health Organization (WHO) in 2008 as a major diagnostic criterion in these three entities and remains in effect, as well as in the International Consensus Classification (ICC) of 2022 [2,3].

In late 2013, mutations in the *CALR* gene were identified. These mutations, which consist of insertions and deletions in exon 9 (the final exon of the gene), are detected in 25–30 % of ET and PMF patients and can be found in up to 60–80 % of *JAK2*-negative patients [1,2].

Several mutations affecting the gene that encodes the thrombopoietin receptor, *MPL*, have also been described in MPNs. These mutations occur primarily in exon 10 of the gene and are reported in about 5 % of PMF and 1–3 % of ET cases [1,2].

Although studies on pH**^–^** MPNs have been carried out in Europe and America, these disorders have a low global incidence, ranging from 1–5 cases per 100,000 inhabitants per year [2,3]. In Ecuador, there is a near-total absence of local data or published studies on pH**^–^** MPNs, which highlights the importance of investigating their frequency, diagnosis, and treatment in the country.

This study is based on information from Ecuadorian patients diagnosed with pH**^–^** MPNs from several health institutions, using data collected between 2014 and 2023. The objective is to provide a local perspective on pH**^–^** MPNs in terms of frequency, clinical presentation, diagnosis and disease evolution, using appropriate statistical methods.

## Methods

This is a descriptive, observational, longitudinal, and multicenter study. Data collection was carried out by hematologists from several healthcare institutions in Ecuador between 2014 and 2023: SOLCA-Guayaquil, Quality of Care (Guayaquil), SOLCA-Quito, “José Carrasco Arteaga” Hospital (Cuenca), and Hospital “Teodoro Maldonado Carbo” (Guayaquil). The study cohort comprised 111 patients diagnosed with BCR-ABL1-negative myeloproliferative neoplasms (MPNs), specifically polycythemia vera (PV), essential thrombocythemia (ET), primary myelofibrosis (PMF), or MPN-unclassifiable (MPN-U). Diagnoses were established according to the World Health Organization (WHO) criteria in effect at the time of each patient's initial presentation [2]. Patients who did not meet the WHO/ICC 2022 diagnostic criteria were excluded [2,3]. Data were collected using an anonymized form on a validated registry platform for this purpose (www.mpn-ecuador.com). The study was approved by the inter-institutional Ethics Committee.

Patient records included data from the date of diagnosis until death or loss to follow-up. Exclusion criteria included the absence of available bone marrow samples or missing information in the electronic registry. Histopathological analyses were performed by trained hematopathologists (FH, PS) who evaluated bone marrow biopsies when reclassification was necessary.

Epidemiological and clinical data were collected, along with information on treatments, complications, and disease progression. Statistical results are presented mainly in terms of frequencies and percentages, while quantitative variables were analyzed using univariate statistics with measures of central tendency and dispersion. A p-value of <0.05 was considered statistically significant.

Data analysis included the creation of cross-tabulation frequency tables, descriptive graphs, survival models, and Multiple Correspondence Analysis (MCA) to enable a more comprehensive exploration using multivariate visualizations. Statistical processing and analysis were conducted using R software (version 4.4.2 for Windows), available at www.r-project.org.

## Analysis and results

### Epidemiology

Among the 111 patients, the most frequent pH**^–^** MPN subtype was PV in 51 cases (46 %), followed by ET in 46 cases (41.4 %). Eleven patients (10 %) were diagnosed with PMF, of which one was in the prefibrotic stage (PMF-P) and the others had established fibrosis (PMF-F). Additionally, three cases (2.7 %) were unclassifiable pH**^–^** MPN (MPN-U). A higher proportion of male patients was observed overall (*n* = 62; 55.85 %), in particular 74.5 % (*n* = 38) patients with PV were male. Conversely, females predominated among patients with ET and PMF, with 28 (60.9 %) and six (54.5 %) cases respectively. Eighty-four patients (75.67 %) were aged 50 years or older, with a predominant subgroup of 54 patients (48.65 %) aged 65 years or older. PV cases were more prevalent in this older group, while ET was more common in patients under 50 years old. No specific age distribution pattern was observed in PMF cases. Regarding habits, six patients (5.4 %) reported alcohol consumption, and 11 patients (9.9 %) were smokers (Tables 1 and 2).

**Table 1:** Frequency of myeloproliferative neoplasms by group.

MPN	n	%
PV	51	45.9
ET	46	41.5
PMF-F	10	9
PMF-P	1	0.9
MPN-U	3	2.7
Total	111	100

MPN: Myeloproliferative neoplasms; PV: Polycythemia vera; ET: Essential thrombocythemia; PMF-P: Primary myelofibrosis prefibrotic stage; PMF-F: Primary myelofibrosis fibrotic stage; MPN-U: Unclassifiable MPN.

**Table 2:** Distribution of myeloproliferative neoplasms by age.

Age	n	%
18–30 years	4	3.61
31–50 years	23	20.72
51–65 years	30	27.02
Over 65 years	54	48.65
Total	111	100

### Comorbidities and clinical presentation

Regarding comorbidities, most patients (*n* = 62; 56 %) had at least one concurrent medical condition. The most common were cardiovascular diseases (*n* = 36; 32.43 %), followed by thyroid disorders (*n* = 13; 11.7 %) and diabetes mellitus (*n* = 10; 9 %).

In terms of clinical symptoms, the most frequently reported were fatigue (*n* = 52; 46.84 %), pruritus (*n* = 29; 26.12 %), weight loss (*n* = 20; 18 %), bone pain (*n* = 11; 9.9 %), diaphoresis (*n* = 10; 9 %), fever (*n* = 5; 4.5 %), migraine (*n* = 1; 0.9 %). The median spleen size of the cohort was 14 cm (range: 9–25 cm), with 61.26 % (*n* = 68) having splenomegaly.

### Peripheral blood and bone marrow biopsy

Blood biometry showed hemoglobin greater than 16 g/dL in 40 cases (36.1 %), anemia in 15 (13.5 %) patients, leukocytosis greater than 10×10^9^/L in 31 (27.9 %) patients, and thrombocytosis in 56 (50.46 %) cases. Serum lactate dehydrogenase (LDH) levels were elevated in 49 patients (44.1 %). Peripheral blood analysis revealed circulating blasts (<5 %) in six patients (5.4 %), while leukoerythroblastosis was observed in eleven cases (9.9 %). Additionally, dacryocytes were identified in the peripheral smears of nine patients (8.1 %).

Bone marrow biopsy findings revealed that the majority of patients exhibited hypercellularity (mean: 59.7 %). Iron deposition was not reported in 81 (72.97 %) patients. Of the remaining 27 % only two (6.7 %) had increased iron levels.

Fibrosis, assessed using Gomori’s histochemical technique [4], was reported in 50 patients (45 %), in the majority, the reticulin fiber network was preserved (MF: 0) (*n* = 43; 86 %). Fibrosis Grade 1 (MF:1) was found in three patients (6 %), Grade 2 (MF:2) in none, and Grade 3 (MF:3) in four patients (8 %). Immunohistochemistry was performed in 31 patients (27.93 %), with CD34 being the most used marker. A cytogenetic study, performed in only 12 patients (10.8 %), detected monosomy 8 in one patient (8.33 %).

### Presence of JAK2 V617F mutation

*JAK2* V617F mutational analysis was performed in 88 (74.8 %) of the patients with the mutation identified in 50 (56.82 %) cases. The highest prevalence was in the ET group (*n* = 24; 52.2 %), followed by PV (*n* = 21; 41.2 %). However, the specific *JAK2* V617F allele burden was not quantified for any of the cases in this registry (Table 3).

**Table 3:** *JAK2* V617F mutation by group.

*JAK2* V617F	Present	Absent	Not performed
PV	21 (41.2 %)	10 (19.6 %)	20 (39.2 %)
ET	24 (52.2 %)	12 (26.1 %)	10 (21.7 %)
PMF	3 (30.0 %)	4 (40.0 %)	4 (30.0 %)
MPN-U	1 (33.3 %)	2 (66.7 %)	0 (0 %)

PV: Polycythemia vera; ET: Essential thrombocythemia; PMF: Primary myelofibrosis; MPN-U: Unclassifiable myeloproliferative neoplasm.

### Treatment

The most used treatment in all MPN groups was hydroxyurea (*n* = 85; 76.6 %), followed by aspirin (*n* = 55; 49.54 %). Ruxolitinib was used in 10 patients (9 %). Approximately half of the patients with PV and ET (*n* = 25 and 22 cases, respectively) were treated with aspirin. Phlebotomy was used in 15 (29.4 %) PV cases and in one (2.17 %) ET patient. Ruxolitinib was administered in three patients with PV, one with ET and six with PMF. Two PMF patients with established fibrosis underwent allogeneic hematopoietic stem cell transplantation. Only one patient in the entire cohort underwent splenectomy. Other treatments were used in 16 % of patients, with clopidogrel being the most frequently prescribed.

### Complications

Thrombotic complications were recorded in seven cases (6.3 %), while hemorrhagic complications occurred in nine patients (8.1 %). Thrombotic events were observed in two cases (4 %) with PV and four cases (8.7 %) with ET, in addition to one patient (33.3 %) with MPN-U. Hemorrhagic complications occurred in two patients (4 %) with PV, one case (2.2 %) with ET, two cases (18.2 %) with PMF, and two cases (66.6 %) with MPN-U.

### Disease progression

Progression/transformation was detected in seven patients (6.3 %); five (4.5 %) with fibrotic progression and two cases (1.8 %) with leukemic transformation. Of the 51 patients with PV, three (5.9 %) progressed to secondary myelofibrosis; while of the 46 cases with ET, two (4.3 %) progressed to post-thrombocythemic myelofibrosis. Among the 11 patients with PMF, one (9 %) case progressed to acute myeloid leukemia as did one (33.3 %) of the three cases of MPN-U.

### Survival

**Figure. 1:**
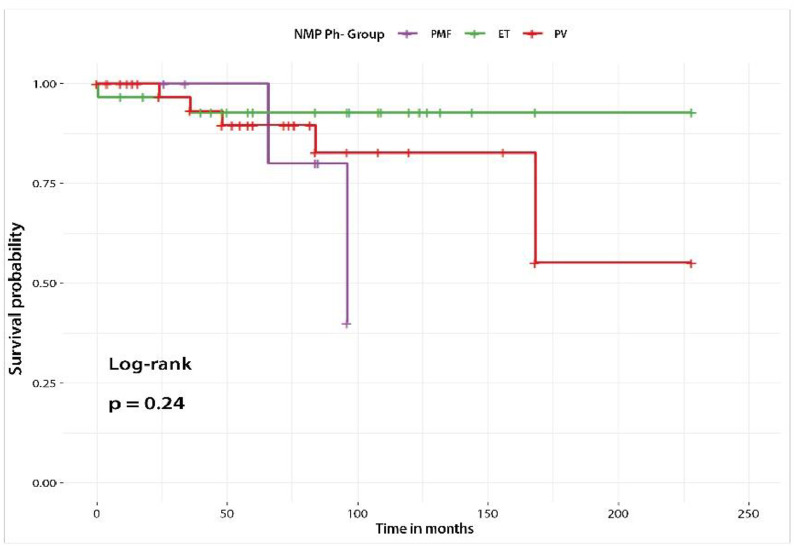
Survival curves for each group of Philadelphia-negative chronic myeloproliferative neoplasms.

The median overall survival was 69 months (range: 0–228). Eleven patients (9.9 %) died during the study. Survival analysis using Kaplan-Meier curves and the log-rank test showed no statistically significant differences between the three major groups (Figure 1). However, due to the smaller sample size of the PMF group, its survival curve declined more rapidly.

**Figure. 2:**
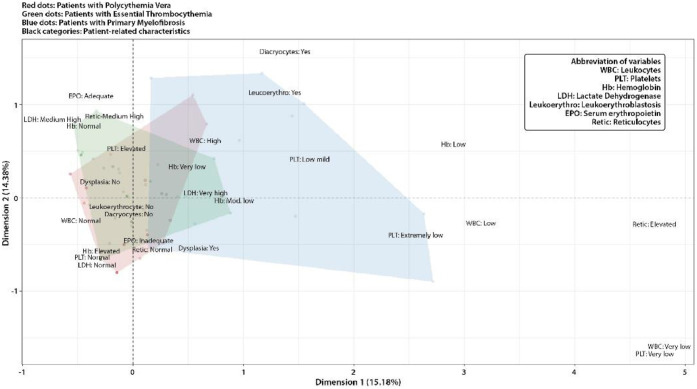
Multiple Correspondence Analysis (MCA): Relationships between characteristics and patients.

A Multiple Correspondence Analysis (MCA) was also performed, including clinical laboratory variables and hematological abnormalities. The first two axes were represented in the MCA, and the area of dispersion of the points corresponding to each pH**^–^** MPN subgroup was delimited by a shaded polygon to simplify visualization. When exploring the frequency tables to support the categories shown in the graph, it was observed that patients with PMF tended to present leukoerythroblasts, dysplasias, high LDH levels and low hemoglobin. This group demonstrated more diverse and high-risk characteristics. Patients with PV showed an association with elevated hemoglobin and inadequate erythropoietin, while those with ET were characterized by thrombocytosis, with the other clinical parameters remaining at normal levels in most of the patients. Although the distribution of the characteristics according to the variables were generally as expected, the total inertia captured by the plot was 29.6 %. The identification of specific trends in each group proved consistent and effective for clinicopathological characterization according to WHO/ICC 2022 criteria [2,3] (Figure 2).

## Discussion

In this study, data on pH**^–^** MPNs patients from five hospital centers in Ecuador were reported and analyzed comparatively with other countries in the region (Central and South America).

The reviewed studies report higher prevalences of ET and PV compared to PMF. In Ecuador, PV was the most frequent (46 %), followed by ET (42 %), which aligns with findings from Cuba, Mexico, Costa Rica, Brazil, Argentina, Colombia, and Chile, where prevalences range from 38–55 % [5, 6, 7, 8, 9, 10, 11, 12, 13,15, 16, 17, 18]. Peru, in contrast, reported a higher incidence of PMF [14].

Regarding age distribution, most studies concurred with our findings: pH**^–^** MPNs predominantly affect patients over 50 years old, with a peak incidence in those over 65 years in Ecuador, a pattern also reported in Chile and Brazil [5,6,11, 12, 13].

In respect to habits, 5–10 % of patients reported smoking or alcoholism in the present study and of the comorbidities, cardiovascular diseases (32 %) were the most frequently reported, with no results being found in the other countries comparing this variable.

It is important to highlight the underuse of complementary histochemical and immunohistochemical techniques. Histochemical staining should be performed on all bone marrow biopsies with suspected pH**^–^** MPN in order to evaluate fibrosis due to its importance in differential diagnosis (ET versus PMF) and prognosis (PV) [4,19,20] although recent studies have explored the application of artificial intelligence (machine learning) directly on Hematoxylin and Eosin-stained images, potentially eliminating the need for additional staining [21].

Molecular testing for the *JAK2* V617F mutation is essential for the classification and prognostic evaluation of pH**^–^** MPNs; however, it was not consistently available to all patients [7, 8, 9, 10, 11, 12, 13]. Nationwide screening is limited by the low number of hospitals and laboratories offering *JAK2* V617F, *CALR*, and *MPL* mutation testing, and by the limited financial resources of patients. Among those tested, the *JAK2* V617F mutation was identified in 56.8 % of cases, a figure comparable to findings from Argentina (57 %), Chile (61 %), and Brazil (62 %) [5,6,11, 12, 13,15,16]. *CALR* and *MPL* mutations were less accessible and mostly reported in studies from Chile, Argentina, and Brazil [6,11, 12, 13,15,16].

In the setting of this study, triple-negative cases often face the additional limitation of not undergoing NGS to detect mutations such as *ASXL1, EZH2, SRSF2, IDH1, IDH2, U2AF1* Q517 among others which are important to confirm clonality and assess prognostic implications [16].

The most frequent complications in the present study were thrombotic events (6.3 %) and hemorrhagic events (8.1 %), which are less common than those reported in the region: Chile (29 %), Colombia (14.7 %), and Brazil (12 %) [5–8,11, 12, 13].

The rate of leukemic transformation was low across the region, with similar figures to Brazil (2.8 %) [12,13]. Costa Rica reported the lowest progression rates [17].

Hydroxyurea, with or without aspirin, was the treatment most frequently used across the region. Notably, the use of JAK2 inhibitors in PMF patients is increasing in Brazil and Argentina [11, 12, 13,15, 16].

In Ecuador, access to a broad therapeutic arsenal remains limited. Several drugs are either inaccessible or lack Ministry of Health authorization, including pegylated interferon alpha (for PV), anagrelide (for ET), and JAK inhibitors such as fedratinib and pacritinib (for PMF patients).

Five-year survival in Ecuador is approximately 90 %, like outcomes reported in Chile and other countries in the region [5,6,14,16].

## Conclusions

Although the study has several limitations, such as selection bias resulting from the limited participation of most health-related institutions in the country, these data provide an initial diagnostic overview of the clinical-pathological and epidemiological status of pH**^–^** MPNs in the Ecuadorean population. The findings highlight the prevalence of PV and ET as the most common forms of pH**^–^** MPNs, with a notable presence of the *JAK2* V617F mutation in these subgroups. The predominant treatment was hydroxyurea. The observed survival across the different types of pH**^–^** MPNs showed no marked variability.

These results reveal a relatively homogeneous epidemiological profile across the Latin American region and underscore the need for more multicenter studies to better characterize pH**^–^** MPNs in Ecuador and the region in order to optimize diagnostic and treatment strategies.

## Data availability

The data that support the findings of this study are available from the corresponding author upon reasonable request.

## Conflicts of interest

The author declares no conflicts of interest.
